# Small bowel obstruction due to encircling of fallopian tube: A curious case report

**DOI:** 10.1016/j.ijscr.2023.108471

**Published:** 2023-07-07

**Authors:** Vadivel Niroshan, Suntharamoorthy Iyer Thuraisamy Sarma, Santhirasegaram Theivaagar, Pushparatnam Abiharan

**Affiliations:** a Professorial Surgical Unit, Teaching Hospital, Jaffna, Sri Lanka; b Department of Surgery, Faculty of Medicine, University of Jaffna, Sri Lanka

**Keywords:** Small bowel obstruction, Fallopian tube, Laparotomy, Gangrenous bowel loops, Salpingectomy, Case report

## Abstract

**Introduction and importance:**

Intestinal obstruction is a common surgical emergency encountered almost in every casualty. Though adhesions, hernias and malignancies are the common causes of obstruction, various articles describe unusual causes of intestinal obstruction which needs timely surgical interventions to prevent morbidity and mortality.

**Case presentation:**

In this case report we present the history of a 50 year old sub-fertile woman who presented with features of intestinal obstruction and confirmed radiologically with both plain x-ray and computed tomography. After conservative management and as the imaging didn't show the cause of obstruction, exploratory laparotomy was performed. There we found have encircling of left fallopian tube around mid-ileum with gangrenous part. Left salphingectomy and bowel resection with side-to-side anastomosis resulted in a favorable outcome.

**Clinical discussion:**

Intestinal obstruction can compromise blood flow to bowel loops leading to gangrene, perforation and death.

**Conclusion:**

Awareness, early recognition and timely intervention in intestinal obstruction is mandatory to prevent poor outcomes, especially in cases of unknown cause and not responding to conservative management. The real surgical challenge is not the decision whether to perform surgery, but the decision when and how to perform it.

## Highlights

• Intestinal obstruction is a common surgical emergency encountered almost in every casualty.
• Though adhesions, hernias and malignancies are the common causes of obstruction, various articles describe unusual causes of intestinal obstruction that needs timely surgical interventions to prevent morbidity and mortality.
• Intestinal obstruction can compromise blood flow to bowel loops leading to gangrene, perforation and death.
• Awareness, early recognition and timely intervention in intestinal obstruction is mandatory to prevent poor outcomes, especially in cases of unknown cause and not responding to conservative management.

## 1 Introduction

Intestinal obstruction is a common surgical emergency. Around 20 % of cases with acute abdominal pain are due to intestinal obstruction, mostly small bowel [1,2]. Most common cause of small bowel obstruction is adhesions while colorectal cancer is for large bowel obstruction. Adhesions formed commonly after abdominal surgery as a result of normal wound healing. Timely intervention will prevent strangulation, gangrene or perforation of bowel. Here we present a case report of a patient with small bowel obstruction caused by encircling of left fallopian tube around the small bowel. Our case report has been written according to the SCARE criteria [3].

## 2 Case presentation

A 50 year old sub fertile female presented with history of sudden onset of generalized colicky pain of 1 day duration. It was associated with repeated bouts of non-bilious vomiting and not passing flatus. She didn't have abdominal distension or fever. In the past she was investigated for subfertility and underwent laparoscopic fimbrial cystectomy of the left fallopian tube. She also had undergone open appendicectomy in the past. No other significant past medical history.

**Fig. 1:**
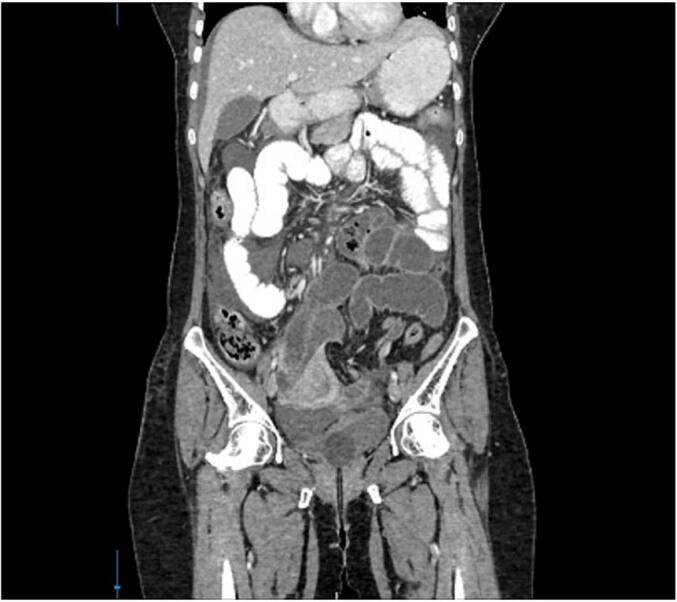
Contrast enhanced CT with small bowel obstruction.

**Fig. 2:**
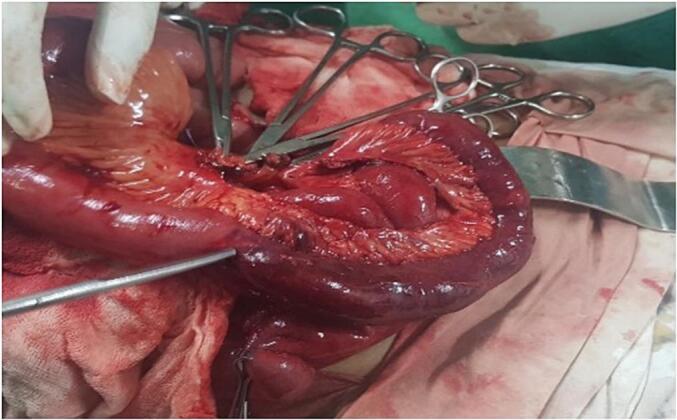
Both healthy and unhealthy(ischaemic) small bowel with artery forceps pointing the fallopian tube encircled part.

On examination she was haemodynamically stable, abdomen was soft with no palpable masses and bowel sounds were sluggish. Digital rectal examination was unremarkable. Her urine HCG (human chorionic gonadotropin) dipstick test was negative. She was investigated in view of intestinal obstruction. Supine x ray of abdomen showed dilated small bowel loops, erect chest x ray was normal and ultrasound sonography shows free fluid in the abdomen with features of small bowel obstruction. After 48 h of conservative management, no improvement in bowel sounds and bowel opening. Contrast Enhanced Computed Tomography (CECT) showed features of small bowel obstruction with the transition point in the mid ileum and cause for obstruction not identified. As the imaging done failed to show the cause of obstruction, it was decided to proceed with explorative laparotomy (Fig. 1, Fig. 2).

Lower midline incision was made and peritoneum was entered. Moderate amount of blood-stained free fluid was found in the cavity. There were no evidences of adhesions that may have caused this obstruction. Impending gangrenous bowel loops were identified and the gangrenous part was encircled by the left fallopian tube. Free end of the fallopian tube strongly adhered to the ovary and broad ligament. In order to release the small bowel without spillage left side salphingectomy has to be done. So, on table consultation was taken from the gynecologist for the salphingectomy. Viability of the bowel loops was tested. Nearly 2 ft of unviable small bowel loop identified and was resected with its mesentery close to the loops. Side to side anastomosis was done using linear staplers. Mesentery defect was closed with absorbable suture material. Her post-operative period was unremarkable, oral intake started on the day 3 with clear fluids and gradually to her normal diet. She was post operatively followed up with duration of 2 weeks, 2 month and 6 month time period to assess her nutritional status, bowel habit and to exclude other features of short bowel syndrome.

## 3 Discussion

Intestinal obstruction is one of the common emergency encountered in surgical field, accounting for nearly 20 % of admissions presenting with acute abdominal pain. In intestinal obstruction more than 75 % are due to small bowel obstruction. Post surgical adhesions are the leading cause of small bowel obstruction while hernias and strictures are next in the list. Colorectal cancer is the leading cause of large bowel obstruction. [1,4]

Cardinal features of obstruction are abdominal pain, vomitting, absolute constipation and abdominal distension. Small bowel obstruction diagnosis is mainly based on a clinical examination and by confirmatory radiological examinations such as plain X ray of the abdominal cavity or computer tomography. Plain supine abdominal x-rays are diagnose small or large intestinal obstruction based on the transverse bands across the bowel and differentiating level of obstruction by small bowel loops located centrally where as large bowel seen peripherally. Erect chest x ray to be done in abdominal tenderness to exclude perforation. Sri Lanka is developing nations and resources are very limited. Though CT is available immediate access is limited; so x-ray was used as a first line investigation.

Contrast enhanced CT important with regard to precise detection of causes of obstruction (other than postoperative adhesions or strangulation) and can be used to differentiate between patients requiring surgeries and patients who can be managed conservatively [5]. Enteric contrast CT was preferred as it is recommended in patients with suspected low-grade small bowel obstruction and it may increase the sensitivity of the diagnosis [6].

In initial management of intestinal obstruction fluid resuscitation and electrolyte replacement with that decompression of proximal bowel by nasogastric tube placement (relieves nausea and vomiting, left to free drain).

Cause for the small bowel obstruction mostly determines the mode of treatment. Period of conservative management can be continued for 48 to 72 h in that majority of cases will resolve [1]. But literatures shows statistically better long term results and lower rate of recurrence in surgical treatment of adhesions compared to conservative management [5]. If the patient develops signs of strangulation such as tachycardia, abdominal tenderness, fever and leukocytosis need early surgical intervention. Constriction around the bowel in band adhesion, need to be resected. Because that heals by fibrosis and cause stricture in future.

A case of intestinal obstruction caused by encircling fallopian tube which is very much similar to our case. In the above case a loop of ileum was encircled by right fallopian tube. Right side salpingo oophorectomy was done to release the bowel loop [7].

Another case where a defect in the broad ligament and fallopian tube acting as a band around the bowel and causing obstruction [8]. In a case reported from India, band was formed by left fallopian tube adhered to the mesentery over the terminal ileum and was causing obstruction [9].

Laparoscopy can be used as a final diagnostic tool to look for the disease and to determine further surgical management whether to continue with laparoscopically or laparoscopic-assisted open approach. In our case, exploratory laparotomy was performed but it would be better with a laparoscopic-assisted open approach. Initially, gastrointestinal obstruction is self-contraindication for laparoscopy, with time change to relative contraindication. Now, the absolute contraindication for laparoscopy in a patient with small bowel obstruction is haemodynamic instability and cardiopulmonary impairment [10]. Relative contraindications to using this technique is insufficient expertise in the management of laparoscopic tools. Laparoscopic technique reduces the rate of complications, shorter hospitalization period and pain compared to a conventional method. Access to the peritoneum via laparotomy associated with higher postoperative adhesions and when using laparoscopy rate of adhesion is reduced by as much as 45 % [5].

## 4 Conclusion

This case highlights the importance of clinical judgement to decide on operative management in small bowel obstruction of unknown etiology not responding to conservative management.

## Consent

Written informed consent was obtained from patient. A copy of the written consent is available for review by the Editor-in-Chief of this journal on request.

## Ethical approval

Ethical approval is waived by the Ethical Review Committee of Teaching Hospital, Jaffna, Sri Lanka.

## Funding

No funding was provided.

## Author contribution

Study concept – V. Niroshan, S.T. Sarma.

Data collection – V.Niroshan, S. Theivaagar, P. Abiharan.

Interpretation - V. Niroshan, S.T. Sarma, S. Theivaagar.

Manuscript preparing - V. Niroshan, S.T. Sarma, P. Abiharan.

## Guarantor

V. Niroshan.

## Registration of research studies

N/A.

## Declaration of competing interest

The authors have no competing interests.

## References

[1] Griffiths S., Glancy D.G. (2020). Intestinal obstruction. Surg (United Kingdom).

[2] Nam Choi H., Dharmawardhane A. (2019). A unique case of small bowel obstruction secondary to internal herniation due to a torted fallopian tube adherent to the sigmoid colon. Int. J. Case Rep. Images.

[3] Agha R.A., Franchi T., Sohrab C., Mathew G., Kirwan A., Thomas A. (2020). The SCARE 2020 guideline: updating consensus surgical case report (SCARE) guidelines. Int. J. Surg..

[4] González-Mesa E., Narbona I., Cohen I., Villegas E., Cuenca C. (2013). Uterine rotation: a cause of intestinal obstruction. Case Rep. Obstet. Gynecol..

[5] Szeliga J., Jackowski M. (2017). Laparoscopy in small bowel obstruction - current status - review. Wideochir Inne Tech Malo Inwazyjne.

[6] Ros P.R., Huprich J.E. (2006 Nov). ACR appropriateness criteria on suspected small-bowel obstruction. J. Am. Coll. Radiol..

[7] Lohani R., Mathur P. (2021). Intestinal obstruction caused by encircling fallopian tube. BMJ Case Rep..

[8] Cameron M., Janakan G., Birch D., Nazir S. (2015). A closed loop obstruction caused by entrapment of the fallopian tube and herniation through the broad ligament. Int. J. Surg. Case Rep..

[9] Rajput D., Kumar U., Gupta A., Kumar N., DS S., Roshan R. (2020). Fallopian tube as a cause of intestinal obstruction: a rare case report with review of literature. Int. J. Res. Med. Sci..

[10] Di Buono G., Ricupati F., Maienza E., Gulotta L., Buscemi S., Agrusa A. (2020). Small bowel obstruction after caesarean section: laparoscopic management. Two case reports. Int. J. Surg. Case Rep..

